# Knowledge scale of Nursing students about sexuality of people with spinal cord injury

**DOI:** 10.1590/0034-7167-2021-0288

**Published:** 2022-08-22

**Authors:** Luana Cristina Hencklein, Daniel Gonçalves Campos, Juliany Lino Gomes Silva, Ruana Luiz Ferreira da Silva, Gabriela Salim Spagnol, Clara Froes de Oliveira Sanfelice, Ana Railka de Souza Oliveira-Kumakura

**Affiliations:** I Universidade Estadual de Campinas. Campinas, São Paulo, Brazil; II Faculdade de Medicina São Leopoldo Mandic. Campinas, São Paulo, Brazil; III Centro Universitário de Jaguariúna. Jaguariúna, São Paulo, Brazil

**Keywords:** Knowledge, Sexuality, Spinal Cord Diseases, Students, Nursing., Conocimiento, Sexualidad, Traumatismos de la Médula Espinal, Estudiantes, Enfermería., Conhecimento, Sexualidade, Traumatismos da Medula Espinhal, Estudantes, Enfermagem.

## Abstract

**Objectives:**

to build, validate and verify the reliability of the Scale of knowledge about sexuality of people with spinal cord injury for nursing students.

**Methods:**

a methodological study, following the steps: 1) Construction based on literature review; 2) Validation of the content with calculation of the Content Validity Index and the Modified Kappa Coefficient; 3) Pre-test with Nursing students; and 4) Measurement of reliability by internal consistency (Cronbach’s alpha).

**Results:**

the first version of the instrument presented 13 items. After validation, the items received values above 0.80 and 0.76 for the Content Validity Index and Modified Kappa Coefficient, and it was suggested to separate three items to contemplate gender-specific aspects. After pre-testing, it was recommended that the writing of two items be revised. The final scale, with 16 items, showed Cronbach’s alpha equal to 0.93.

**Conclusions:**

the constructed scale presented valid content and proved to be reliable for application with nursing students.

## RESUMO

**Objetivos:**

construir, validar e verificar a confiabilidade da Escala de conhecimento sobre sexualidade de pessoas com lesão medular para estudantes de Enfermagem.

**Métodos:**

estudo metodológico, seguindo as etapas: 1) Construção baseada em revisão da literatura; 2) Validação do conteúdo com cálculo do Índice de Validade de Conteúdo e do Coeficiente Kappa Modificado; 3) Pré-teste com estudantes de Enfermagem; e 4) Medida da confiabilidade pela consistência interna (alfa de Cronbach).

**Resultados:**

a primeira versão do instrumento apresentou 13 itens. Após validação, os itens receberam valores acima de 0,80 e 0,76 para o Índice de Validade de Conteúdo e Coeficiente Kappa Modificado, e sugeriu-se separar três itens para contemplar aspectos específicos do sexo. Após pré-teste, recomendou-se a revisão da escrita de dois itens. A escala final, com 16 itens, apresentou alfa de Cronbach igual a 0,93.

**Conclusões:**

a escala construída apresentou conteúdo válido e mostrou-se confiável para aplicação com estudantes de Enfermagem.

## RESUMEN

**Objetivos:**

construir, validar y verificar confiabilidad de Escala de conocimiento sobre sexualidad de personas con lesión medular para estudiantes de Enfermería.

**Métodos:**

estudio metodológico, siguiendo las etapas: 1) Construcción basada en revisión de la literatura; 2) Validez de contenido con cálculo del Índice de Validez de Contenido y Coeficiente Kappa Modificado; 3) Pretest con estudiantes de Enfermería; y 4) Medida de confiabilidad por consistencia interna (alfa de Cronbach).

**Resultados:**

la primera versión del instrumento presentó 13 ítems. Posterior validación, estos recibieron valores arriba de 0,80 y 0,76 para el Índice de Validez de Contenido y Coeficiente Kappa Modificado, y sugerido separar tres ítems para contemplar aspectos específicos del sexo. Posterior pretest, recomendado la revisión de la escrita de dos ítems. La escala final, con 16 ítems, presentó alfa de Cronbach igual a 0,93.

**Conclusiones:**

la escala construida presentó contenido válido y se mostró confiable para aplicación con estudiantes de Enfermería.

## INTRODUCTION

Spinal cord injury (SCI) is defined as damage to the structures contained within the spinal canal, which encompasses the spinal cord, the *conus medullaris*, and the *cauda equina*; it can be traumatic or non-traumatic in origin^(1-2)^. In this context, it is essential to guarantee multi-professional, integral and humanized assistance to the person with spinal cord injury from pre-hospital care to rehabilitation, considering that the alterations of this condition interfere in several physical functions, such as spasticity, lack of sensory capacity, neurogenic bladder, autonomic dysreflexia, erectile dysfunction, among others^(3)^.

The rehabilitation process of the person with SCI involves early care of these changes, and therapeutic interventions should be defined with the objective of improving respiratory mechanics, monitoring the adequate intake of nutrients, ensuring the adaptation to the new conditions related to the vesicular-intestinal system, controlling and preventing complications related to spasticity and autonomic dysreflexia, in addition to considering care related to social, economic and emotional aspects^(4)^. In this context, sexual rehabilitation is necessary in order to contribute to body self-awareness, sexual satisfaction and emotional development, aiming to adapt the individual to his new condition^(5)^.

The multidisciplinary team, especially nurses, are important players in this process, performing actions that must meet the psychosocial, motor, functional and spiritual needs of people with SCI, ensuring that both individuals and their families are cared for in an integral way^(6)^. However, a World Report on Disability shows that many health care professionals need access to knowledge and skills to provide an individualized care plan for people with disabilities, highlighting that undergraduate programs rarely address the changing health of this population^(7)^.

The theme of sexuality is gradually being inserted in the undergraduate curricula of higher education institutions, including in the health area. However, students may not feel prepared to deal with these issues^(8)^. A study on sexuality education for health professionals showed that higher education courses do not offer the necessary content in academic training, and the disciplines focused on this theme and the discussion on human sexuality are still not very comprehensive^(8)^. Thus, the gap in the training process of professionals can have repercussions on their future performance, determining failures in the comprehensive and humanized care of people with SCI.

Still in this regard, a systematic review of qualitative studies analyzed perceptions and experiences with sex, sexuality and relationships of individuals after SCI, and the results showed high dissatisfaction with the care in health education for sexual rehabilitation. Individuals reported that, in general, health professionals are not prepared to address this issue^(9)^. Therefore, it is essential to discuss this theme since the academic formation, so that future professionals can deal with these issues in a respectful and humanized way.

Among health professionals, nurses are the main ones responsible for health education actions, which make it important to include the topic “sexuality” in their training to acquire knowledge. An effective method to overcome the lack of knowledge on this topic is the use of assessment tools for professionals and nursing students on the sexuality of the person with SCI. This directly influences the application of educational interventions focused on promoting teaching, which has an impact on the quality of care offered^(10)^.

However, there are no instruments to assess the knowledge about the sexuality of SCI patients aimed at Nursing professionals and students in the current scientific literature. What is observed are scales that evaluate the knowledge related to the sexuality of individuals with SCI^(11)^.

In view of the above, there is a scarcity of productions in the literature on standardized and applicable instruments for professionals and nursing students to assess their knowledge on the theme of sexuality and rehabilitation of SCI patients. Thus, considering that instruments on the subject can stimulate this discussion, raise weaknesses in teaching and encourage the development of training focused on filling these gaps^(12)^, the objective of this study was to develop a reliable and valid instrument for application in this population.

Therefore, when thinking about the construction of instruments, it is important to highlight that some steps need to be followed to make the construct studied observable and measurable. These are: definition of the conceptual structure, elaboration of the objectives and population, construction of the items and the response scale, selection and organization; structuring of the instrument, judgment by experts; pre-test; measurement of its reliability and validity^(13)^.

## OBJECTIVES

To build, validate and verify the reliability of the Scale of knowledge about sexuality of people with spinal cord injury for nursing students.

## METHODS

### Ethical aspects

This study was approved by the Research Ethics Committee of the State University of Campinas and followed the ethical standards for research involving human beings contained in Resolution 466/12 of the National Health Council. The participation of subjects occurred after signing the Free and Informed Consent Term.

### Study design, time and place

This is a methodological study, with cross-sectional design and quantitative approach, having occurred in four stages: 1) Construction of the Scale of Knowledge about Sexuality of People with Spinal Cord Injury; 2) Validation of content; 3) Pre-test; and 4) Measurement of reliability^(13-17)^.

To guide the testing phase of the research with the target population, the guidelines for observational studies, called strengthening the Reporting of Observational Studies in Epidemiology (STROBE) checklist: cross-sectional studies, were adopted. Data collection occurred in the period from August 2019 to January 2020, with nursing students from two institutions, one public and one private, located in the interior of the state of São Paulo.

### Population or sample; inclusion and exclusion criteria

For content validation, 11 experts were invited after analyzing their resumes on the Lattes Platform. There is no consensus in the literature about the adequate sample size, being recommended from 5 to 20 experts. However, for the adoption of the minimum number, it is important to prioritize the following aspects in the expert’s profile: clinical experience, research on the topic, and knowledge of the conceptual framework addressed^(14-15)^. Therefore, for the current research, the inclusion criteria were listed: experience in assistance or teaching with the population diagnosed with SCI or with the topic “sexuality” of at least two years; and a minimum degree of Master’s in Nursing or Health Sciences. Experts who agreed to participate but did not return the completed instrument within 30 days were excluded from the study.

The literature points out that pre-testing the scale is an effective step in assessing comprehension of the items and can include a sample of 30 to 40 individuals^(16)^ or use the brainstorming technique with a group of at least four people from the lowest stratum of the target population, because once they understand the scale and its items, the more sophisticated stratum will also understand it^(14)^. In this study, this lower stratum refers to students in the first semesters of the course, as opposed to those who attend the final semesters. Thus, the sample of the pre-test phase was selected according to the availability and acceptance of the nursing students to participate in this stage, and they could not have studied the topics of sexuality or SCI during their training, which was questioned prior to participation. It should be noted that this study is part of a larger research whose objective was to evaluate the effect of an educational intervention on the knowledge of nursing students, which justifies the selection of a sample with no knowledge of the theme.

For the reliability measure, Nursing students of any academic period from a public university and a private university were included. The literature recommendation was followed^(17)^ for the inclusion of a minimum sample size of 50 students, with a final sample size of 52. Students who agreed to participate but did not complete all items on the knowledge scale were dropped from the sample.

### Study protocol

The construction of the scale was based on the theoretical foundation developed to establish the variables associated with the sexuality of people with SCI. Thus, the key elements of this phenomenon in the selected public were identified (dimension and operationalization of the construct), based on literature review^(18-19)^. Next, the scale items were formulated and then their difficulty was verified by the research team. At the end, the standardization for the application of the scale was established (which orientations should come in the header so that the student alone would know how to fill out the scale), its evaluation (how the final score would be calculated) and interpretation of the result obtained (the score achieved would represent more or less knowledge about the theme under study).

For Stage 1 of the study, two review studies were used^(11,20)^ which were identified and selected to guide the construction of the scale items on the main changes related to the sexuality of SCI patients. The items to compose the knowledge scale were selected by the frequency of appearance and by the judgment of the research team, since it had clinical experience in providing care to people with SCI and theoretical knowledge on the subject under study. Since several outcomes were assessed in these instrument reviews, we also verified studies focused on recommendations to health professionals to work with the theme in order to list priority changes in the literature and be included in the scale of this research^(9,12)^. Although the present research was directed to nursing students, the researchers decided to include studies that addressed health professionals: first, to expand the selected productions; second, because the idea is to train students still in training, so that in the future, as professionals, they can provide care that is more directed to the real needs of this public.

In the second stage of this study, we proceeded to content validation. This is a fundamental process for the development of any instrument that intends to measure and associate abstract concepts with measurable indicators, defined then as a way to judge if each component element of an instrument is relevant and representative to what is proposed to be evaluated within the theme^(15)^.

Prior contact was made by e-mail with the specialists to pass on information about the objective, methodology, and justification for the development of the instrument. After acceptance, the material for evaluation was sent via e-mail, and a period of one month was set for the content to be appreciated and suggestions to be returned.

For each item of the scale, it was requested the evaluation of the presence or absence of the criteria of clarity and pertinence of psychometrics; and, for the set of items of the scale, it was requested the evaluation of its Amplitude/Breadth^(13,16,21)^. Based on these criteria, they were to assign the following scores: 1. item not representative/not clear OR Scale not comprehensive; 2. item needs major revision to be representative and clear OR Scale needs major revision to be comprehensive; 3. item needs minor revision to be representative and clear OR Scale needs minor revision to be comprehensive; and 4. item is representative and clear OR Scale is comprehensive. Only one round was conducted with the experts; and if they chose options 1 or 2, they were asked to justify the changes or deletion of the items^(13,16,21)^. After returning the instruments, a meeting was held between the research team, and adjustments were made to the scale items according to the experts’ recommendations. It was not necessary to return the material for a new appreciation of them, since the suggestions did not cause changes in the content of the items of the scale.

Next, the pre-test stage was performed. A member of the research team presented the scale and asked the students to initially fill it out, and then the scale was analyzed item by item to allow the observation of their understanding. If any difficulty was identified in understanding the item, it should be adjusted to avoid interpretation problems^(14,22)^. In addition, an instrument was provided for the participating nursing students to evaluate the adequacy and clarity of the scale, globally and of each of its items. Next, the pre-test students were asked to appreciate the type of response scale chosen, i.e., to state whether the four-point Likert scale ranging from “no knowledge” to “excellent knowledge” was clear enough for them to select the answer^(13,16,21)^. Filling out the knowledge scale took about five minutes.

After the pre-test, a new version of the scale was developed in order to contemplate the students’ suggestions. As the modifications made did not alter the content of the items, there was no need to return the material to the experts, and the final version was appreciated by the research team involved.

To complete the verification of the measurement properties, the reliability of the scale was estimated^(16-17)^ through internal consistency analysis, with a sample of 52 students (Step 4). They were verbally approached in classrooms and by institutional e-mail, individually and/or in groups, and invited to participate in the study. An invitation letter was sent by e-mail with a link to Google Forms containing the instrument for analysis. If any student did not have access to the internet to fill out the form, they were offered the opportunity to do so in the printed version in a reserved room after class.

The students who participated in this stage filled out not only the scale that was previously constructed and validated, but also a characterization instrument that contained questions about personal data (name, sex, race/color, marital status, place of birth, origin), educational/institutional data (completion of a technical course in Nursing, other undergraduate course, current undergraduate institution, semester and course period) and information regarding previous contact with teaching methodologies, in addition to a question about participation in any class on sexuality and/or spinal cord injury.

### Analysis of results and statistics

For the analysis of the Content Validity Index (CVI) and the Modified Kappa Coefficient (MKC), the Microsoft Excel for Windows® program was used; and for the Cronbach’s alpha analysis, SPSS, version 23 was used. The MKC was used to evaluate the level of agreement among experts in Step 2 regarding the clarity and pertinence of the items and the amplitude of the scale. Values higher than 0.75 represent excellent agreement; values between 0.75 and 0.40 represent average agreement; and values below 0.40 represent low agreement^(13,16,21)^. In addition, the CVI, another measure to evaluate the percentage of agreement among experts, was adopted. For CVI, values higher than 80% are recommended^(13,16,21)^. In this study, indicators with MKC greater than 0.80 and CVI greater than 80% were considered relevant.

The measurement of the scale’s reliability was examined by analyzing the internal consistency, using Cronbach’s alpha coefficient. Furthermore, it was verified the value of Cronbach’s alpha if each item was deleted, being evidence of satisfactory internal consistency a value greater than or equal to 0.70^(13,16,21)^. To complement the measure, the item-total correlation was presented, whose values equal or above 0.30 are recommended^(23)^.

## RESULTS

### Construction of the scale

During Step 1, the main items were listed to build the scale of knowledge about sexuality of people with SCI for nursing students. These items were identified through reviews conducted in two previous research studies^(11,20)^. Thus, the selected articles were read in full and the main changes in the person with SCI in the field of sexuality that should be known by students were mapped, given the relevance of this knowledge in the comprehensive care of people with SCI.

The scale initially contemplated 13 evaluation items, with four possible answers, in which the current knowledge about the topics was indicated on a Likert-type scale, as follows: 1 - no knowledge; 2 - little knowledge; 3 - good knowledge; and 4 - excellent knowledge. The scale scores could range from 13 to 52 points, with a higher score indicating a higher level of knowledge. Chart 1 shows the results of Stage 1 (construction), as well as those of Stages 2 (content validation) and 3 (pre-test), which will be discussed in depth in the following topics.

**Chart 1:** Summary of the results of the construction, content validation and pre-test of the Knowledge Scale about sexuality in people with spinal cord injury for Nursing students, Campinas, São Paulo, Brazil, 2019

Construction	Content Validation	Pre-test
Title: Evaluation of the Scale of Knowledge about Sexuality of Patients with Spinal Cord Injury	Replace “patients” with “people”.	Maintained
1. Sexual anatomy and physiology	Maintained	Maintained
2. Fertility procedures	Change to “Fertility procedures in cases of spinal cord injury: natural, medication and artificial techniques”.	Maintained
3. Reproductive capacity	Change to “Reproductive capacity of the person with spinal cord injury”.	Maintained
4. Male and female contraception	Separate into “Contraception for the man with spinal cord injury” and “Contraception for the woman with spinal cord injury”.	Maintained
5. Male and female orgasm	Separate into “Orgasm of the man with spinal cord injury” and “Orgasm of the woman with spinal cord injury”.	Maintained
6. Bowel care during sexual activity	Change to “Bowel care for the person with spinal cord injury prior to sexual activity”.	Maintained
7. Bladder care during sexual activity	Change to “Bladder care for the person with spinal cord injury prior to sexual activity”.	Maintained
8. Sexual positions	Modify to “Sexual positions for the person with spinal cord injury”.	Maintained
9. Methods for achieving erection	Change to “Methods to achieve erection in men with spinal cord injury”.	Change to “Methods to achieve erection in the person with spinal cord injury”.
10. Methods to get vaginal lubrication	Modify to “Methods to achieve vaginal lubrication in women with spinal cord injury”.	Change to “Methods to achieve vaginal lubrication in the person with spinal cord injury”.
11. Autonomic dysreflexia	Change to “Autonomic dysreflexia in people with spinal cord injury”.	Maintained
12. Male and female sexuality	Separate into “Sexuality of the man with spinal cord injury” and “Sexuality of the woman with spinal cord injury”.	Maintained
13. Techniques and resources to work with sexual rehabilitation	Change to “Techniques and resources to work with the sexual rehabilitation of the person with spinal cord injury”.	Maintained

### Content Validation

Five specialists participated in this stage, all PhDs in Nursing, aged 29 to 44 years, residents in the states of Ceará (3), Piauí (1) and Rio de Janeiro (1), with professional training time ranging from 8 to 22 years and teaching time from 3 to 14 years.

In Stage 2, only Question 3 presented lower CVI and MKC values, being revised according to suggestions (Table 1). Despite agreeing with the clarity and relevance of all items, the experts suggested the separation of three items (4, 5, and 12) (Table 1).

**Table 1:** Values of the Content Validity Index and Modified Kappa Coefficient for the set of items, according to the criteria of clarity and pertinence; and for the scale of knowledge about sexuality of people with spinal cord injury, according to the criterion of amplitude, Campinas, São Paulo, Brazil, 2019

Items	Clarity		Pertinence		Amplitude
	CVI	MKC	CVI	MKC	
1	1	1	1	1	-
2	1	1	1	1	-
3	0.8	0.76	1	1	-
4	1	1	1	1	-
5	1	1	1	1	-
6	1	1	1	1	-
7	1	1	1	1	-
8	1	1	1	1	-
9	1	1	1	1	-
10	1	1	1	1	-
11	1	1	1	1	-
12	1	1	1	1	-
13	1	1	1	1	-
Scale	-	-	-	-	1

*CVI - Content Validity Index; MKC - Modified Kappa Coefficient.*

All suggestions and considerations of the experts were incorporated into the final version, which allowed the adaptation of the scale’s content. The following changes were made: replacement of the word “patient” by “person”; specification of the sentences that related to the situation of SCI patients; and separation of the conditions that were specific to men and women, to improve the clarity of the expressions. After analysis and discussion by the research team, all suggestions were incorporated in full. Therefore, the final scale included 16 items about the sexuality of SCI patients, in a four-point Likert-type format, scoring from 16 to 64 points.

### Pre-test

Based on the analysis of the answers of the five students who participated in the pre-test brainstorming format and the data contained in the data collection instrument, there were modifications in the questions about penile erection and vaginal lubrication, because the students referred that the scale items should consider people with gender identity different from their biological sex assigned at birth. Thus, the items “methods for achieving vaginal lubrication in women with spinal cord injury” and “methods for achieving erection in men with spinal cord injury” had their nouns “man” and “woman” changed to “people”. Unfortunately, it was not possible to change these nouns in all items, which makes some answers impossible for the non-binary population that does not identify with any gender.

In general, the five students scored positive aspects of the scale, such as the fact that it was adequate to assess knowledge (5/5 students) and that it did not take much time to complete (average time was 5.2 minutes). As for the items, they said that they were clear, that is, easy to understand and with direct sentences (4/5 students), that the quantity was adequate (1/5 students), and that the information was enlightening and organized in a logical sequence (4/5 students). For the answer format, they placed that it was adequate and with enough options (5/5 students).

### Reliability Measurement

Fifty-two nursing students participated in this stage, with a mean age of 22.48 years (SD = 3.59), ranging from 18 to 35 years. Most of them were female (88.46%), single (88.46%) and from Campinas (48.08%). They belonged to two universities, one public (67.31%) and the other private (32.69%), and were predominantly enrolled in the 7th (25%), 10th (21.15%) and 5th (19.23%) semesters of the course. About 53.85% had taken a SCI class and 96.15% reported that they had not taken a class on the sexuality of the person with SCI.

Regarding the measure of internal consistency, the final scale with 16 items showed Cronbach’s alpha equal to 0.93 (Table 2), which shows high reliability. The exclusion of “Item 1 - Sexual anatomy and physiology” increased the alpha value to 0.94. As for the item-total correlation, it was higher than 0.30 for all items of the scale, showing that they are related to the sexuality domain of SCI patients. Thus, after the reliability analysis, we do not recommend the exclusion of any item.

**Table 2:** Reliability analysis of the Scale of knowledge about sexuality in people with spinal cord injury and its items, Campinas, São Paulo, Brazil, 2019

Items	Item-total correlation	Cronbach's alpha, if item deleted	Cronbach's alpha for the scale
Anatomy and sexual physiology	0.32	0.94	
Fertility procedures in cases of medullar injury: natural techniques, medication and artificial	0.69	0.92	
Reproductive capacity of the person with medullar injury	0.58	0.92	
Contraception of the man with medullar injury	0.62	0.92	
Contraception of the woman with medullar injury	0.63	0.92	
Orgasm of the man with medullar injury	0.72	0.92	
Orgasm of the woman with medullar injury	0.74	0.92	
Intestinal care of the person with medullar injury prior to sexual activity	0.65	0.92	
Bladder care of the person with a medullar injury prior to sexual activity	0.64	0.92	
Sexual positions for the person with medullar injury	0.67	0.92	
Methods to achieve an erection in the person with medullar injury	0.71	0.92	
Methods to achieve vaginal lubrication in the person with medullar injury	0.62	0.92	
Autonomic dysreflexia in people with medullar injuries	0.72	0.92	
Sexuality of the man with medullar injury	0.69	0.92	
Sexuality of the woman with medullar injury	0.75	0.92	
Techniques and resources to work with the sexual rehabilitation of the person with medullar injury	0.78	0.92	
Scale			0.93

Table 3 presents the profile of the students’ knowledge about the study theme. Only for “Item 1 - Sexual Anatomy and Physiology”, the students reported having good (59.6%) and excellent (9.6%) knowledge. For the other items of the scale about fertility procedures, reproductive capacity, contraception, orgasm, urinary and bowel care, sexual positions, erection, vaginal lubrication, autonomic dysreflexia, sexuality in general and techniques to work with sexual rehabilitation, there was a predominance of “no knowledge” to “little knowledge”.

**Table 3:** Nursing students’ knowledge about the sexuality of people with spinal cord injury, in percentage, Campinas, São Paulo, Brazil, 2019

Items	No knowledge (%)	Little knowledge (%)	Good knowledge (%)	Excellent knowledge (%)
Anatomy and sexual physiology	13.46	17.31	59.62	9.62
Fertility procedures in cases of medullar injury: natural techniques, medication and artificial	65.38	32.69	1.92	0
Reproductive capacity of the person with medullar injury	50	44.23	5.77	0
Contraception of the man with medullar injury	63.46	34.62	1.92	0
Contraception of the woman with medullar injury	55.77	42.31	1.92	0
Orgasm of the man with medullar injury	80.77	15.38	3.85	0
Orgasm of the woman with medullar injury	78.85	17.31	3.85	0
Intestinal care of the person with medullar injury prior to sexual activity	65.38	30.77	3.85	0
Bladder care of the person with a medullar injury prior to sexual activity	67.31	23.08	9.62	0
Sexual positions for the person with medullar injury	80.77	17.31	1.92	0
Methods to achieve an erection in the person with medullar injury	88.46	7.69	3.85	0
Methods to achieve vaginal lubrication in the person with medullar injury	80.77	17.31	1.92	0
Autonomic dysreflexia in people with medullar injuries	78.85	17.31	3.85	0
Sexuality of the man with medullar injury	76.92	19.23	3.85	0
Sexuality of the woman with medullar injury	75	23.08	1.92	0
Techniques and resources to work with the sexual rehabilitation of the person with medullar injury	88.46	7.69	3.85	0

## DISCUSSION

This study presented the stages of construction, content validation, pre-test and reliability measurement of the Scale of knowledge about sexuality of people with spinal cord injury for nursing students. Nowadays, the approach to issues such as those addressed in this research is necessary for the understanding of students’ knowledge about human sexuality and spinal cord injury, in order to facilitate the identification of gaps related to the theme, facilitate the teaching and learning process and, above all, provide a better basis for care.

Other studies discuss this contribution by assessing the lack of knowledge of professionals about the construct being investigated and defining interventions to try to reduce this deficit, so that they can contribute to the provision of care^(12,24)^. Therefore, the steps taken so far have helped achieve this goal for nursing students.

However, it is not always possible to cover all the items on the subject, being primordial the attempt to address the most representative aspects of the theme. In this case, the contents that served as a basis for the construction of this scale allowed the identification of aspects of the sexuality of people who, with the occurrence of SCI, start to present changes that need to be addressed in the context of rehabilitation, which should be started since the establishment of the injury^(11-20)^.

As for content validity and reliability, the scale showed good indicators according to the literature, which recommends strict compliance with the construction, review by a committee of experts, in addition to conducting the pre-test^(13,16,21)^.

That said, expert assessment is fundamental to recognize the target audience of the instrument, as well as to identify if the content can meet the reality of the sexuality of the person with SCI^(13)^. This instrument followed the recommendation to fulfill this step and verified the importance of the experts’ suggestions for the content validation process.

The assessment of the psychometric properties of a new instrument, according to recommendations in the literature, should be carried out prior to application in the population for which it is intended. The execution of a pre-test is essential to verify the understanding of the items, make adjustments, and detect inconsistencies^(16)^. The instrument of this research went through a pre-test with Nursing students; and, despite not requiring significant changes after its application, the results obtained were considered fundamental. The good CVI and MKC values as well as the satisfactory Cronbach’s alpha (0.93) attest to the content validity and reliability of the scale^(16-17)^.

Just like other studies^(9-10,25)^, the current instrument tried to address the main changes in the sexuality of the person with SCI that need to be discussed in the training of health professionals.

When analyzing the knowledge of the sample that participated in the stage of verification of the scale’s reliability measure, it is noted that for “Item 1 - Sexual anatomy and physiology”, considered the broadest on the subject, students reported having good knowledge. However, for the other items, the knowledge was classified between none and little, which shows the need to work and deepen issues related to the theme during the students’ training. Moreover, these findings may encourage the trainer to think about different teaching strategies that can be used to address this issue, either in undergraduate or continuing education.

Therefore, there is a need to address and deepen the subject in teaching, as well as to incorporate techniques for the performance and care of future nursing professionals who will work in the care of people with SCI. It is believed that the qualification of the teaching of this theme will positively impact the quality of life of the person with SCI, favoring the experience of a safe and satisfactory sexuality. The coverage of issues such as gender, sexual and body orientation and self-esteem, sexual desire, sexual function and satisfaction, relationship with the partner and sexual education are fundamental, because they are outcomes assessed in validated instruments on the subject^(11)^.

The search for validated instruments for use with health professionals pointed to only one study, whose objective was to study attitudes, approach, knowledge and comfort regarding sexuality and disability. However, the instrument was not validated to be used in the rehabilitation process, even though sexuality and disability are being increasingly recognized in this process^(26)^.

A recent study applied the scale built in the present research to assess the effect of an educational intervention on the knowledge of nursing students about the topic of sexuality in people with SCI. This study identified that the topics of less knowledge before the educational intervention were related to male and female orgasm, sexual positions, and methods to achieve erection, methods to achieve vaginal lubrication, autonomic dysreflexia and techniques to work on rehabilitation. Notwithstanding the one-off use of two simulation scenarios, students’ knowledge of items relating to autonomic dysreflexia, techniques for working on rehabilitation, fertility procedures, and male contraception improved. However, the differences between the means before and after the application of the intervention were smaller than for the other school items, which reinforces the need for continuity of education on the subject^(27)^. Thus, the scale developed and validated in this study showed promise for use with nursing students to address the sexuality of men and women with SCI.

### Study limitations

The scale went through a content validation stage that included only one professional category, however, it is recommended to use a multiprofessional sample given the subjectivity of the phenomenon studied. The psychometric properties evaluated in the instrument may vary according to the sample used for data collection, which, in the case of this study, were undergraduate nursing students from two institutions in the same region of the country, so the insertion of another scenario, institution or sample may generate other results on the analyzed properties. As for the measurement properties examined, only content and reliability validation was performed, and other analyses can be added. Next, the pre-test was performed with a small sample of individuals. Although the final scale excludes non-binary people by using the nouns “men” and “women”, it is essential to include this subject in teaching-learning. Furthermore, one can think that studies on sexuality present difficulties at the methodological level, because it is a subject of an intimate nature still permeated with prejudices and taboos.

### Contributions to the field of Nursing, Health or Public Policy

It is expected that the application of this scale can identify the gaps in knowledge of nursing students on the topic of sexuality of people with SCI. Furthermore, that this scale be used as an instrument to evaluate the effect of applying different teaching methodologies and/or strategies with the aim of improving the acquisition of knowledge on the subject.

The importance of the recognition, by nursing students, of the elements of sexuality of people with SCI is reiterated. When this is well worked out, there is a strategy capable of qualifying the care and favoring the experience of a full sexuality of this population.

## CONCLUSIONS

The process of analysis of the measurement properties showed that the constructed scale presented evidence of content validity and reliability for application with a sample of Nursing students.

## SUPPLEMENTARY MATERIAL

The manuscript has research data available at https://data.scielo.org/dataset.xhtml?persistentId=doi:10.48331/scielodata.ETWEHW.

0034-7167-reben-75-06-e20210288-sup01
