# Supplementary material for: Knowledge scale of Nursing students about sexuality of people with spinal cord injury
Source: Rev Bras Enferm. 2022 Aug 22;75(6):e20210288. doi: 10.1590/0034-7167-2021-0288 (PMC9749499; doi:10.1590/0034-7167-2021-0288)
Supplement: 0034-7167-reben-75-06-e20210288-sup01 [file 0034-7167-reben-75-06-e20210288-sup01.pdf]

README

Título do conjunto de dados: Escala de conhecimento sobre sexualidade de pessoas com lesão medular para estudantes de enfermagem

Pesquisador principal: Ana Railka de Souza Oliveira-Kumakura
Universidade Estadual de Campina
arailka@unicamp.br

Data de coleta dos dados: A coleta de dados ocorreu no período de agosto de 2019 a janeiro de 2020.

Visão geral dos dados e arquivos (breve descrição dos dados que cada arquivo contém, data de criação de cada arquivo e como eles se relacionam entre si, etc): O arquivo trata-se de duas planilhas do Excel. A primeira planilha contém os dados da validação de conteudo, etapa 2 da pesquisa, a qual foi completada em novembro de 2019. A segunda planilha refere-se a fase 4 da pesquisa, a qual foi executada em dezembro de 2019 e janeiro de 2020 e permitiu a verificação da Medida da confiabilidade da Escala de Conhecimento sobre sexualidade de pessoas com lesão medular

Descrição dos métodos de coleta ou geração dos dados: Foi realizado contato prévio por e-mail com os especialistas para repasse das informações sobre o objetivo, metodologia e justificativa para a elaboração do instrumento. Para cada item da escala, solicitou-se a avaliação da presença ou ausência dos critérios de clareza e pertinência da psicometria e para o conjunto de itens da escala, solicitou-se a avaliação da sua Amplitude/Abrangência. Com base nesses critérios, solicitou-se a atribuição das seguintes notas: 1. Item não representativo/não claro OU Escala não abrangente; 2. Item necessita de grande revisão para ser representativo e claro OU Escala necessita grande revisão para ser abrangente; 3. Item necessita de pequena revisão para ser representativo e claro OU Escala necessita pouca revisão para ser abrangente; e 4. Item representativo e claro Escala abrangente. Foi realizada apenas uma rodada com os especialistas e caso eles escolhessem as opções 1 ou 2, eram solicitadas justificativas para as alterações ou exclusão dos itens. Para completar a verificação das propriedades de medidas, foi estimada a confiabilidade da escala, por meio da análise da consistência interna, com uma

amostra de 52 estudantes (Etapa 4). Os estudantes que participaram desta etapa, além de preencherem a escala que foi construída e validada previamente, também preencheram um instrumento de caracterização que continha perguntas sobre dados pessoais (nome, sexo, raça/cor, estado civil, naturalidade, procedência), dados de formação/instituição (realização de curso técnico de enfermagem, outra graduação, instituição atual da graduação, semestre e período do curso) e informações referentes ao contato prévio com metodologias de ensino, além de se questionar sobre a participação em alguma aula sobre sexualidade e/ou lesão medular.

Descrição dos métodos usados para o processamento dos dados: Para a análise do Índice de Validade de Conteúdo (IVC) e do Coeficiente Kappa modificado (CKM) foi utilizado o programa Microsoft Excel para Windows® e para a realização das análises do alfa de Cronbach foi utilizado o SPSS, versão 23.

Informações específicas dos dados (lista de variáveis, unidades de medida, definições de códigos ou símbolos, calibragem de equipamentos, etc): Nas duas planilhas constam os dados referentes as variáveis sociodemográfica de cada participante. No caso da Planilha 1, referente aos especialistas, coletou-se infomaçoes sobre sua experiência profissional e de ensino, participação em grupo de pesquisa e assistência aos pacientes com lesão medular. NO caso da planilha 2, para os estudantes, foi verificada a experiência previa com diferentes estratégias de ensino e com o conteudo de sexualidade e lesão medular.
